# Impact of dietary level and ratio of n-6 and n-3 fatty acids on disease progression and mRNA expression of immune and inflammatory markers in Atlantic salmon (*Salmo salar*) challenged with *Paramoeba perurans*

**DOI:** 10.7717/peerj.12028

**Published:** 2021-08-31

**Authors:** Chandrasekar Selvam, Mark D. Powell, Nina S. Liland, Grethe Rosenlund, Nini H. Sissener

**Affiliations:** 1 Institute of Marine Research, Bergen, Norway; 2 Central Marine Fisheries Research Institute, Kochi, India; 3 Marineholmen RAS Lab AS & University of Bergen, Bergen, Norway; 4 Skretting ARC, Stavanger, Norway

**Keywords:** Atlantic salmon, n-6/n-3 FA ratio, Paramoeba perurans, AGD, Immune response, Dietary fatty acids

## Abstract

The aim of the study was to investigate the influence of dietary level and ratio of n-6/n-3 fatty acids (FA) on growth, disease progression and expression of immune and inflammatory markers in Atlantic salmon (*Salmo salar*) following challenge with *Paramoeba perurans*. Fish (80 g) were fed four different diets with different ratios of n-6/n-3 FA; at 1.3, 2.4 and 6.0 and one diet with ratio of 1.3 combined with a higher level of n-3 FA and n-6 FA. The diet with the n-6/n-3 FA ratio of 6.0 was included to ensure potential n-6 FA effects were revealed, while the three other diets were more commercially relevant n-6/n-3 FA ratios and levels. After a pre-feeding period of 3 months, fish from each diet regime were challenged with a standardized laboratory challenge using a clonal culture of *P. perurans* at the concentration of 1,000 cells L^−1^. The subsequent development of the disease was monitored (by gross gill score), and sampling conducted before challenge and at weekly sampling points for 5 weeks post-challenge. Challenge with *P. perurans* did not have a significant impact on the growth of the fish during the challenge period, but fish given the feed with the highest n-6/n-3 FA ratio had reduced growth compared to the other groups. Total gill score for all surfaces showed a significant increase with time, reaching a maximum at 21 days post-challenge and declined thereafter, irrespective of diet groups. Challenge with *P. perurans* influenced the mRNA expression of examined genes involved in immune and inflammatory response (TNF-α, iNOS, IL4-13b, GATA-3, IL-1β, p53, COX2 and PGE_2_-EP4), but diet did not influence the gene expression. In conclusion, an increase in dietary n-6/n-3 FA ratio influenced the growth of Atlantic salmon challenged with *P. perurans*; however, it did not alter the mRNA expression of immune genes or progression of the disease.

## Introduction

The rapid growth of the aquaculture industry has raised concern about its sustainability and environmental impacts. One concern is the high dependency on marine ingredients such as fish meal and fish oil for aquafeeds, which could increase the pressure on the already vulnerable global fisheries. In many aquaculture-grown fish species, there has been a large shift in dietary composition in the last two decades, with a replacement of the majority of marine ingredients with other available ingredients, such as plant oils and proteins. Several studies in Atlantic salmon have demonstrated the possibility of partial or complete replacement of fish oil with plant oils without any adverse effects on growth, feed utilization, and survival of the fish (Hixson et al., 2017; Katan et al., 2019; Liland et al., 2013; Sissener et al., 2016b; Torstensen et al., 2005; Turchini, Torstensen & Ng, 2009). However, there are some limitations in using plant oils. For example, they completely lack the very long-chained polyunsaturated fatty acids (>20C fatty acids, LC-PUFA, *e.g.*, eicosapentaenoic acid, 20:5n-3, EPA; docosahexaenoic acid, 22:6n-3, DHA; arachidonic acid, 20:4n-6, ARA). These fatty acids (FA) are considered crucial for many physiological processes, and are thus important for optimal growth and health of the fish. Additionally, most plant oils contain high linoleic acid (18:2n-6) than α-linolenic acid (18:3n-3). Thus, increased plant oil in fish feed at the expense of fish oil greatly affects the FA profile in the feeds, with decreasing LC-PUFA levels and increases in both absolute content of n-6 FA as well as dietary n-6/n-3 ratio.

As dietary FA directly influences membrane lipid composition, manipulating dietary n-6 and n-3 FA will alter the ratio of ARA and EPA in cell membranes. This can, in turn, alter the production of eicosanoids and modulate the inflammatory and immune responses in fish (Bell et al., 1991; Bell, Tocher & Sargent, 1994). As in mammals, eicosanoids are key mediators of inflammation in fish and play a central role in immune regulation (Bruce German, Bruckner & Kinsella, 1986; Rowley et al., 1995; Rowley et al., 2012). Although this is a complex system (Araujo et al., 2019), eicosanoids derived from the n-6 family, such as leukotriene B_4_ (LTB_4_) and prostaglandin E_2_ (PGE_2_) are generally considered to have a greater ability to promote pro-inflammatory responses than eicosanoids from the n-3 family, which are considered to have more anti-inflammatory properties (Lands, 1992). However, there are studies that have reported the inhibitory effects of PGE_2_ on classical proinflammatory cytokines such as TNF-α and IL-1β in human whole blood culture (Miles, Allen & Calder, 2002) and fish cell line models (Fast, Ross & Johnson, 2005; Furne et al., 2013). Further, it has been described that PGE_2_ has both pro-inflammatory and anti-inflammatory roles and that some lipoxins derived from ARA, especially lipoxin A4, may be important for switching off inflammation (Calder, 2009; Liu et al., 2017). Although ARA is the preferred substrate for the main enzymes cyclooxygenase (COX) and lipoxygenase (LOX) in eicosanoid production pathways, an increased EPA levels in cell membrane can competitively inhibit the production of n-6 derived eicosanoids (Bell et al., 1996a; Bell et al., 1996b). Similarly, a study in human cell models (HUVEC) reported the high production of COX metabolites when exposing the cells to ARA resulted in increased production. However, the high levels of COX metabolites were suppressed when ARA was combined with DHA, ALA (Araujo et al., 2019). Thus, the ratio between these FA is important for subsequent eicosanoids production. The balance between n-6 and n-3 FA in the diet is therefore important for a healthy inflammatory-and immune response (Calder, 2008; Simopoulos, 2002), and the potential modulation of immune and inflammatory responses by dietary n-6/n-3 ratio remains in the interest of research. In fish, information about the relationship between dietary n-6/n-3 ratio, immune responses and specific disease resistance is limited and contradictory. In Atlantic salmon, variable results on health impact of increased n-6/n-3 ratios have been reported, ranging from no apparent negative effects (Andresen et al., 2019; Bransden, Carter & Nichols, 2003; Gjøen et al., 2005; Grisdale-Helland et al., 2002; Hundal et al., 2021), to significantly increased mortality following transportation induced stress (Bell et al., 1991), reduced resistance to infection (Martinez-Rubio et al., 2012; Thompson, Tatner & Henderson, 1996), effects on the humoral immunity and expression of immune related genes (Caballero-Solares et al., 2017; Seierstad et al., 2009), and increased production of n-6 FA derived prostaglandins (Hundal et al., 2021). The contradicting reports regarding the link between dietary lipids and immune regulation in fish remain unclear and it may be due to interactions between environment, pathogens and fish.

Amoebic gill disease (AGD) caused by the protozoan *Paramoeba perurans* (syn. *Neoparamoeba perurans*; Feehan et al., 2013), is established in many salmon producing areas and poses a major challenge for salmon aquaculture industry. AGD and other infections represent a source of progressive stress for salmon (Nowak & Archibald, 2018; Robledo et al., 2020). A few studies on functional diets and AGD infection are reported (Bridle et al., 2005; Mullins et al., 2020; Powell et al., 2007). However, to our knowledge, there are no controlled studies on the relation between dietary fatty acids and gill infections. Nevertheless, there are dietary studies on fatty acids where the fish happened to be affected with AGD during the trial, indicating a relation between the severity of the disease with the dietary n-6/n-3 ratio (Glencross et al., 2014; Sissener et al., 2016b). While AGD is the result of an ecto-parasitic infection, there is evidence of immune and inflammatory responses in the infected gills (Marcos-Lopez et al., 2018; Marcos-Lopez et al., 2017; Pennacchi et al., 2014), as well as a systemic physiological compromise (Powell et al., 2008), which means an effect of dietary FAs is plausible. Therefore, the present study was designed to determine the effects of both absolute levels of dietary n-3 and n-6 FA, and the n-6/n-3 ratios on disease progression, growth, and mRNA expression of selected immune and inflammatory markers during amoebic gill infection in Atlantic salmon.

## Materials and Methods

### Experimental diets

The experimental diets were produced by Skretting ARC (Stavanger, Norway). The formulation for the experimental diets is shown in Table 1 (previously published in Hundal et al., 2020). A total of four different diets were formulated to contain different absolute contents of n-6 and n-3 FA and n-6/n-3 ratios from 1.0–6.0. The n-3 FAs were kept constant for the first three diets and constituted approximately 8% of total FA. Roughly half of the n-3 FAs were provided as EPA + DHA (10 g/kg of EPA + DHA in the final feed) to meet the minimum requirement (Rosenlund et al., 2016). In the same three diets, the n-6 FA levels were adjusted to create n-6/n-3 ratios of 1, 2 and 6. The diet with the n-6/n-3 ratio of 6.0 was included to make sure potential n-6 effects were revealed, while the three other diets had more commercially relevant n- 6/n-3 ratios of averaging 0.9 (Sele et al., 2018). The fourth diet was formulated to contain double the amount of n-3 FA as compared to first three diets (20 g/kg of EPA + DHA in the final feed), but with an n-6/n-3 ratio of 1, like the first diet. The experimental diets are referred in the text according to their dietary n-6/n-3 ratios and named diet 1, diet 2, diet 6 and diet 1H (diet 1H, due to its higher absolute contents of n-3 and n-6 FA compared to diet 1) (previously published in Hundal et al., 2020). All the experimental diets were produced from a common dry meal mixture and differed only in the combination of oils used to adjust the n-3 and n-6 FA content of the extruded pellets. Proximate and FA composition of the experimental diets (3 and 4 mm pellet) have been described previously in detail by Hundal et al. (2020) and are also presented in Table 2 (4 mm pellet) for a better understanding of the current study.

**Table 1:** Feed formulation in g/100 g of the four diets used in the challenge trial (4 mm pellet size). Previously published in Hundal et al. (2020).

	Diet 1	Diet 2	Diet 6	Diet 1H
Wheat	7.2	7.2	7.2	7.2
Soya protein concentrate	28.2	28.2	28.2	28.2
Sunflower meal	6.0	6.0	6.0	6.0
Wheat gluten	18.0	18.0	18.0	18.0
Faba beans dehulled	2.0	2.0	2.0	2.0
Fish meal North Atlantic	10.0	10.0	10.0	10.0
Linseed oil	1.2	1.2	1.3	2.7
Sunflower oil	0.6	4.8	19.3	5.1
Olive oil	12.2	7.7	0.0	1.6
Coconut oil	0.7	1.0	0.3	0.0
Fish oil North Atlantic	0.0	0.0	3.9	4.9
Fish oil Capelin	10.0	10.0	0.0	10.5
Premixes	3.7	3.7	3.7	3.7
Yttrium	0.1	0.1	0.1	0.1

**Note:**

Diet 1/Diet 2/Diet 6/Diet 1H, diet codes are set according to dietary n-6/n-3 ratio. The final diet is labelled 1H due to its higher absolute contents of n-3 and n-6 compared to the first diet.

**Table 2:** Analysed dietary proximate (g/100 g) and fatty acid composition (% of total FA), total FA (mg/g) of the four diets used in the challenge trial (4 mm pellet). Previously published in Hundal et al. (2020).

	Diet 1	Diet 2	Diet 6	Diet 1H
Proximate composition g/100 g				
Lipid	29.8	30.0	28.6	28.8
Protein	44.5	44.1	44.5	44.8
Ash	5.1	5.2	5.5	5.4
Fatty acids (% of total FA)				
ΣSFA	19.7	19.7	16.6	19.8
12:0	1.3	1.7	0.4	0.1
14:0	3.3	3.5	1.5	4.4
16:0	11.2	10.4	9.4	11.5
18:0	2.5	2.6	3.7	2.7
ΣMUFA	59.4	52.2	27.3	43.5
16:1n-7	3.8	3.7	1.5	5.1
18:1n-7	2.2	2.0	1.1	2.0
18:1n-9	37.1	30.1	23.1	18.2
20:1n-9	6.4	6.4	0.7	7.0
22:1n-11	7.7	7.7	0.7	8.5
Σn-6	11.1	18.2	46.9	18.4
18:2n-6	10.8	17.9	46.7	17.6
20:4n-6 (ARA)	0.1	0.1	0.1	0.2
Σn-3	7.7	7.7	7.8	15.1
18:3n-3	2.9	2.9	3.1	5.7
18:4n-3	0.5	0.5	0.4	0.9
20:5n-3 (EPA)	2.4	2.4	2.0	4.4
22:6n-3 (DHA)	1.5	1.5	1.8	3.1
EPA + DHA	3.9	3.9	3.8	7.5
ΣPUFA	20.9	28.1	56.1	36.8
n-6/n-3	1.4	2.4	6.1	1.2
Sum FA (mg/g)	270.7	272.7	296.5	255.3

**Note:**

Diet 1/Diet 2/Diet 6/Diet 1H, diet codes are set according to dietary n-6/n-3 ratio. The final diet is labelled 1H due to its higher absolute contents of n-3 and n-6 compared to the first diet; FA, fatty acid; SFA, saturated fatty acids; MUFA, monounsaturated fatty acids; EPA, eicosapentaenoic acid; DHA, docosahexaenoic acid; PUFA, polyunsaturated fatty acids.

### Pre-feeding

The feeding trial was started with a pre-feeding period and performed at Skretting ARC Research station at Lerang, Norway (December 2017). Atlantic salmon (SalmoBreed, Erfjord, Stamfisk AS) with a mean weight of 80 g were randomly distributed to four large circular tanks (3 m inner diameter and water holding capacity of around 7,000 L), 735 individuals in each tank. The tanks were supplied with flow through sea water (25–27ppt) at 8 °C and 24 h light photoperiod was maintained throughout the pre-feeding period. The fish were fed to satiation with experimental diets (one tank per diet; 3 mm pellet size, proximate and FA composition published in Hundal et al., 2020) and the pre-feeding period lasted for about 4 months (till March 2018) to stabilize FA composition of tissues. During the pre-feeding period, fish were examined routinely and no signs of AGD related infection or any other disease conditions were found.

### Amoeba

The amoebae (*P. perurans*) for the challenge experiment were obtained and used under the license from ILAB (Industrial and aquatic laboratory, Bergen Norway). The original amoeba clone (ES301013 C2) was isolated from an AGD outbreak in Sotra in 2013 by ILAB, according to methods described by Morrison, Crosbie & Nowak, 2004 and 18S rRNA gene PCR method (Young et al., 2008) were used to detect and identify the species and confirmed as *P. perurans*. From this, an *in vitro* monoclonal culture was established as described by Crosbie et al. (2012). The amoebae were sub-cultured regularly and occasional PCR tests were carried out to confirm the presence of *P. perurans* (Young et al., 2008). The virulence of the amoebae was also assessed regularly, as described by Collins et al. (2017) and the same amoeba clone were previously used for other challenge experiments and a reliable and reproducible infection developing AGD to mean gill score of 2 within 21 days (Rosenlund, 2017). For this study, the amoebae were cultured in liquid media (0.1% Malt Yeast Seawater Broth) in plastic culture flasks maintained at 15 °C and isolated by scraping before counting using a hemocytometer.

### Challenge experiment and sampling

A challenge experiment was performed at the ILAB challenge facility, Bergen, Norway. After the pre-feeding period, fish-(250 fish from each diet group) were transported to ILAB by truck designed for transportation of live fish (Jarle Tveiten Transport, Tørrvikbygd, Norway), and maintained full strength salinity (34 ppt) and water temperature 8 °C. After arrival, fish were distributed into the experimental tanks (500 L); three technical replicates for each diet group (three tanks containing 60 fish/diet) were used as challenge groups and one tank for each diet group was assigned as a negative control group. The average weight and length of the fish assigned for each experiment tank were 335.4 ± 20.3 g (mean ± SD) and 28.6 ± 0.6 cm (mean), respectively. Prior to the challenge, the fish were acclimated to the tank conditions for 2 weeks. During this period, the water temperature was gradually increased from 8 to 13 °C and 34 ppt salinity was maintained. The experimental tanks were supplied with flow-through filtered seawater (34 ppt) at 13 °C and 12:12 light:dark photoperiod was maintained. The fish were fed to satiation with the experimental diets (4 mm pellet) throughout the challenge study. Water quality parameters were regularly monitored, and standard husbandry procedures were followed as per the guidelines from ILAB.

The fish in the challenge groups were exposed to *P. perurans* trophozoites at a concentration of approximately 1,000 cells L^−1^. This challenge concentration has been established and is recognized to induce disease at a moderate rate at 13 °C (Downes et al., 2017; Pennacchi et al., 2016; Rosenlund, 2017). Prior to inoculation with amoebae, the water flow to the tanks (including negative control tanks) was stopped. The appropriate amount of amoebae isolate was added to each tank and the fish were maintained under static water conditions for 60 min with constant aeration before the water flow was reinstated. The behavior of the fish was carefully observed throughout the entire exposure, and in the immediate hours thereafter.

A pre-challenge sample point (week 0, prior to the addition of amoebae) was performed, followed by weekly sampling point throughout the 5-week trial. At the first four samplings (0 dpc; days of post challenge, 7 dpc, 14 dpc and 21 dpc), 10 fish per tank were sampled and at the last two samplings, 7 fish per tank were sampled. At each sampling event, fish from the tank were randomly removed by dip net and euthanized by an overdose of Finquel vet. (Tricainmesilat, 100 mg L^−1^). Weight and length of the fish were recorded. All gill arches of the fish were visually assessed for the presence and severity of lesions and scored on a scale from 0 to 5, with 0 representing no signs of infection and 5 representing a severe AGD infection, according to (Taylor et al., 2009b). To reduce blood contamination of gill samples, fish were first bled by caudal venous puncture. Gills from the left second gill arch was carefully dissected out and filaments from the apex region of the gill arch were flash frozen in liquid N_2_ and stored at −80 °C for gene expression analysis. The remaining part of the gill arch was fixed in a 10% neutral buffered formalin solution (VWR^®^) and processed for histology (3–5 µm sections stained with H&E). The third left gill arch was dissected, and flash frozen in frozen in liquid N_2_ and stored in −80 °C for FA profile analysis.

### mRNA expression in gill tissue

To study the effect of diets on gill mRNA expression, samples from 0 dpc (pre-challenge), 21 dpc (maximum gill score) and 28 dpc (recovery phase) samplings were used (n = 6 individual fish per tank). The selected target genes for mRNA expression analysis include pro-inflammatory cytokines (TNF 1α, IFN γ, IL1β, IL4-13b), genes involved in eicosanoids production pathway (PGE2-EP4, COX-2), cellular stress markers (HSP70, HSP90), tumor suppressor protein p53 (p53), the inducible isoform of nitric oxide synthase (iNOS) and T-Cell-specific transcription factor GATA-3 (GATA-3). The procedures for RNA extraction, reverse transcription and qPCR followed are described in detail in Hundal et al. (2021). In brief, total mRNA was extracted from gill tissue using EZ1 RNA Universal Tissue Kit (Qiagen, Crawley, UK) and the Bio Robot EZ1 according to the manufacturer’s instructions. Quality and integrity of RNA were assessed with the NanoDrop ND-1000 UV–Vis Spectrophotometer (NanoDrop Technologies, Wilmington, DE, USA) and the Agilent 2100 Bioanalyzer (Agilent Technologies, Palo Alto, CA, USA). A two-step, real-time RT-PCR protocol was followed to assess the mRNA transcriptional levels of the selected target genes. The stability of the reference genes (β-actin and EF1ab) and mean normalized expression of the target genes were calculated using CFX Maestro software (Bio-Rad CFX maestro version 1.1, Bio-Rad laboratories, Hercules, CA). The primer sequences of the selected target genes as well as the reference genes are given in Table 3 (Invitrogen, Life Technologies, Waltham, MA, USA).

**Table 3:** Fatty acid composition (percentage of total fatty acids) of gills from Atlantic salmon fed different diets at 21 days of post-challenge with *P. perurans*.

Target genes	Forward primers	Reverse primers	Accession number
PGE_2_-EP4	CTGATTATGATGCACAAGCGGTTCA	GTTTACAAAAATCCGCAGCACCAAAG	KM519440
COX2	GATCGCTGGAAGGGTGGCTG	GCCAGCTCTGTCTCTCCTGTGAGGT	AGKD04000045
TNF-α	GTGTATGTGGGAGCAGTGTT	GAAGCCTGTTCTCTGTGACT	NM_001123617
IL-1 β	GCTGGAGAGTGCTGTGGAAGAAC	CGTAGACAGGTTCAAATGCACTTTGTG	AY617117
iNOS	GCTACACGACATGAAACACCCAGAGTT	GGACATCCTGGACATAGACCTTTGG	Benedicenti et al. (2019)
IL4-13b	CTCAATGGAGGTTTGGAGTTTCAGG	TGCAGTTGGTTGGATGAAACTTATTGT	HG794525
GATA3	ACCCAAGCGACGACTGTCTG	GGTGAGAGGTCGGTTGATATTGTG	XM_014153208
p53	CTTGGGAGGGATATGATAATTTC	AGGGTAGAGATGGAGGGCTG	XM_014195886
Hsp70	CATCGACTTCTACACCTCCATCAC	CTGAAGAGGTCGGAACACATCTC	AJ632154
Hsp90	GTGTGAACAATGGGAAATGGAACA	CAGCGTGCATGTTATGTTGCA	NM_001173702.1
β-actin	CCAAAGCCAACAGGGAGAA	AGGGACAACACTGCCTGGAT	BG933897
EF1αb	TGCCCCTCCAGGATGTCTAC	CACGGCCCACAGGTACTG	AF321836

**Note:**

Results are means ± SD (for challenged group *n* = 3, for non-challenged group. 3 fish per diet). P-values of two-way ANOVA are presented for factors ‘diet’, ‘AGD challenge’ and interaction between diet and AGD challenge. ns, not statistically significant (*p* > 0.05). Different superscript letters within an individual row denote statistically significant differences in fatty acid content according to Tukey’s multiple comparison test. FA, fatty acid; ΣSFA, sum of saturated fatty acids; ΣMUFA, sum of monounsaturated fatty acids; EPA, eicosapentaenoic acid; DHA, docosahexaenoic acid; PUFA, polyunsaturated fatty acids. Diet 1/Diet 2/Diet 6/Diet 1H, diet codes are set according to dietary n-6/n-3 ratio. The final diet is labelled 1H due to its higher absolute contents of n-3 and n-6 compared to the first diet. PGE_2_-EP4, prostaglandin E2-EP4 receptor; COX2, cyclooxygenase2; TNF-α, tumour necrosis factor- α; IL-1β, induction of interleukin-1β; iNOS, inducible nitric oxide synthase; IL413-b, interleukin 4/13b; GATA3, transcription factor GATA binding protein3; p53, tumor suppressor protein p53; Hsp70, Heat shock protein 70; Hsp90, Heat shock protein 90; β-actin, Beta-actin; EF1 αb, Elongation factor 1 alpha B.

### Fatty acid composition

The FA composition of gill tissue was determined according to the method by (Lie & Lambertsen, 1991) as previously described in detail (Jordal et al., 2005; Sissener et al., 2016a), using gas liquid chromatography (Scion 436-GC, Scion Instruments, UK). The FA were identified by their retention times using a standard mixture of methyl esters (Nu-Chek-Prep, Elysian, MN, USA) and the FA composition (area %) was determined. Quantification of FA was done using 19:0 as an internal standard and integration of peak areas was done using software Chromeleon^®^ version 7.2 (Thermo Scientific, Waltham, MA, USA). The FA composition of the feed was analysed by Skretting ARC, according to the method described by Rosenlund et al. (2016).

### Ethics statement

The feeding trial and subsequent disease challenge was conducted according to the guidelines of the Norwegian State Commission for Laboratory Animals and the protocol for the challenge experiment was approved by the National food safety authority (Mattilsynet, Norway) under the permit number 14333.

### Statistics

Statistical analysis was performed using the software Statistica 13.4 (Statsoft Inc., Tulsa, OK, USA) and GraphPad Prism version 8.0 (Graphpad Software Inc., San Diago, CA, USA). Data were tested for normality and homogeneity of variance using a Kolomogorov–Smirnov test and Shapiro-wilk test. Data from gene expression analysis were log transformed for statistical analysis. All data were tested for tank effect using nested ANOVA (tanks nested under groups), and no significant difference was observed between the replicates. The data were subjected to a two-way analysis of variance (ANOVA), with diet and days post-challenge as the two factors. Only in the cases where a significant effect was observed within a factor, one-way ANOVA followed by Tukey’s multiple comparison were performed for each factor separately. For all statistical tests, *p*-values < 0.05 were considered significant. All results are expressed as mean ± standard deviation (SD).

## Results

### Growth

**Figure 1:**
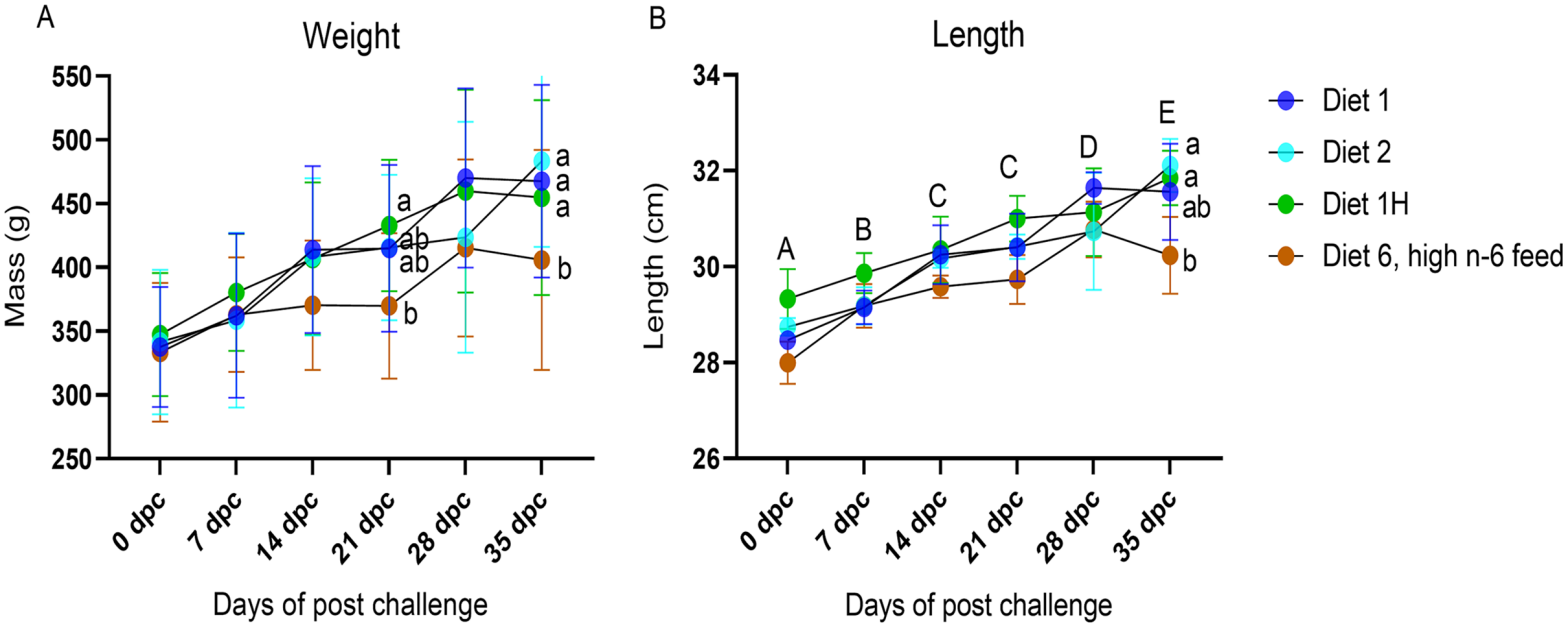
Weight (A) and Length (B) of Atlantic salmon fed different level and ratio of n-6 and n-3 FA and challenged with *P. perurans*. Data presented as mean with standard deviation (*n* = 3). Two-way ANOVA followed by Tukey’s Multiple comparison was performed for factors diet and AGD challenge. Capital letters (A, B) indicate significant differences between the time-points (*p* < 0.05) and small letters (a, b) indicate significant differences between the dietary group (*p* < 0 .05) detected in nested one-way ANOVA. Diet 1/Diet 2/Diet 6/Diet 1H, diet codes are set according to dietary n-6/n-3 ratio. The final diet is labelled 1H due to its higher absolute contents of n-3 and n-6 compared to the first diet.

The body weight and length were registered for all fish sampled at each sampling point (Fig. 1; Table S1). No differences were found in the mean individual start body weight (335.4 ± 20.3 g; mean ± SD) or length (28.6 ± 0.6 cm; mean ± SD) of the fish assigned to each experimental tank for the challenge trial. No dietary effects on weight or length were seen in the non-challenged fish. In the challenged fish, diet had a significant effect on the weight and length of the fish. The fish fed with the highest n-6/n-3 ratio (6:1, diet 6) had a significantly reduced weight compared to the other dietary groups (diet 1, diet 2 & diet 1H) at 21 dpc and 35 dpc (*p* = 0.0004 and 0.038, respectively; Fig. 1). Similarly, length of the fish were also significantly affected at 35 dpc (*p* = 0.017, Fig. 1) in the group fed with the highest n-6/n-3 ratio (6:1, diet 6) compared to other dietary groups. There was a borderline negative interaction on weight of the fish between time after AGD exposure and dietary treatments (*p* = 0.07, Fig. 1). No mortality was observed during the experiment.

### Gill pathology

**Figure 2:**
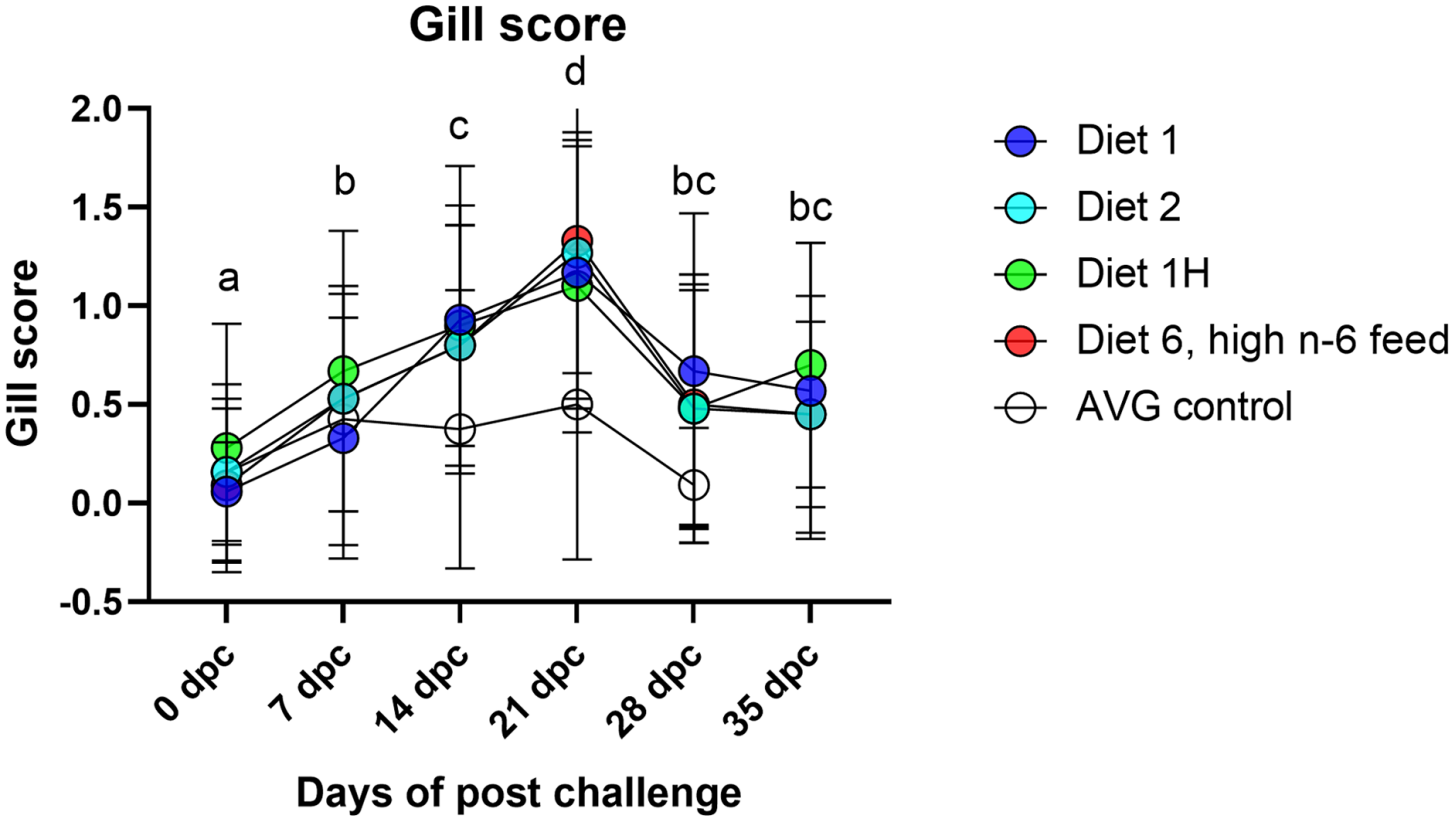
Gill score of Atlantic salmon fed different level and ratio of n-6 and n-3 FA and challenged with *P. perurans*. Data presented as mean with standard deviation (*n* = 3). Two-way ANOVA followed by Tukey’s Multiple comparison was performed for factors diet and AGD challenge. Different letters indicate the significant differences between the time-points (*p* < 0.05). No significant effects between diet groups were detected at any time points. Diet 1/Diet 2/Diet 6/Diet 1H, diet codes are set according to dietary n-6/n-3 ratio. The final diet is labelled 1H due to its higher absolute contents of n-3 and n-6 compared to the first diet. AVG control, unchallenged fish.

**Figure 3:**
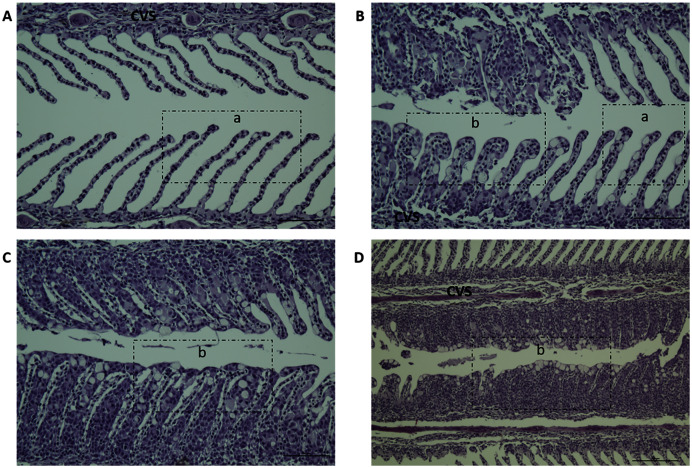
Histopathology representative examples of *P. perurans* challenged Atlantic salmon gills at 21 dpc. Lowercase letter (a) indicates the healthy gill and (b) indicates the hyperplastic lesions with fused lamellae and the stratified layer of epithelial tissue at the lesions surface. The central venous sinus is denoted as CVS; bar = 50 µm (A, B, C); bar = 100 µm (D).

Gill scores and severity of gill lesions were recorded at all the sampling points (*n* = 10 per tank, three tanks per diet group challenged fish, one tank per diet in non-challenged control-groups) to assess how different dietary n-6/n-3 ratios influence the gill response and health of the fish upon infection with *P. perurans*. No signs of AGD were visible upon gross examination of the gills of the uninfected control fish at any of the sampling points. In the challenged fish, the gill score for all surfaces and severity of gill lesions showed a significant increase with time, reaching a maximum at 21 dpc, declining after that (Fig. 2; Table S2). At the pre-challenge sampling point (0 dpc), fish showed no signs of AGD. At 7 dpc, the gill scores had increased to 0.52 ± 0.03 (mean ± SD) in the challenged fish, displaying only a few focal, raised white patches on the gills. Thereafter, gill scores further increased at 14 dpc (0.84 ± 0.13), reaching a maximum at 21 dpc (1.21 ± 0.11; mean ± SD; Fig. 2). There was a clear temporal change in the severity of gross pathology, with distinguishable AGD-like lesions across the gills at 21 dpc. At sampling points 28 dpc and 35 dpc, mean gill scores were decreased to 0.53 ± 0.28 and 0.53 ± 0.08 (mean ± SD), respectively (Fig. 2). There were significant differences in gill score between the different sampling points after infection (*p* < 0.0001; Fig. 2), but no dietary effects were found during challenge period. Histologically, no classical characteristic signs of AGD lesions were observed and no amoebae were associated with the lesions. However, a few fish at 21 dpc, showed a mild hyperplastic lesion (ht) with fused adjacent lamellae and stratified layer of epithelial tissue at the lesions surface (Fig. 3). At 21 dpc, three fish were positive for *P. perurans* by qPCR (Pharmaq analytiq, Bergen, Norway).

### Fatty acid composition of the gills

The FA composition of the gills was analyzed for both challenged and non-challenged fish at 21 dpc, where we observed the maximum gill score (Table 4). The FA composition of the gills largely reflected the dietary FA composition. In both challenged and non-challenged fish, the percentage of ∑SFA (~25% of total FA) in the gills showed no significant difference between the dietary groups despite diet 6 having lower absolute dietary SFA contents than the other dietary groups. The percentage of ∑MUFA, however, was significantly different between dietary groups and reflected the dietary MUFA content. In both challenged and non-challenged fish, total n-6 FA content in the gills increased with increasing dietary n-6/n-3 ratio. The fish fed diet 2 had slightly elevated level of some of the n-6 FA (20:3n-6, 20:4n-6) compared to diet 1H, despite both diets containing equal amounts of n-6 FA. In the challenged fish, the ARA content was significantly increased with increasing dietary n-6/n-3 ratio (diets 1, 2 and 6), and for the dietary group 1H, ARA content was intermediate between the dietary groups 1 and 2. In the non-challenged fish, only the fish fed diet 6 showed a higher ARA in gill tissue compared to the other dietary groups. The EPA levels were significantly reduced with increasing dietary n-6/n-3 ratio in the diets (diets 1, 2 and 6). However, levels of DHA were similar between all dietary groups, despite differences in dietary DHA content. Further, AGD challenge did not significantly influence the levels of these FA (EPA and DHA). There was a significant interaction effect between challenge and diet on total n-6 FA in gill, challenged fish fed diet 6 had significantly reduced total n-6 FA (*p* = 0.0356) compared to the other dietary groups. This was due to a reduction in 18:2n-6 (*p* = 0.019) in the challenged fish, while other n-6 FA showed no significant differences between challenged and non-challenged fish. There also was a significant interaction between diet and challenge for ARA, appearing to have a response in the opposite direction than 18:2n-6 in diet group 6 when challenged.

**Table 4:** Nucleotide sequence of primers used in qPCR for mRNA expression analysis of target and house-keeping genes.

	Challenged group				Non-challenged group				Two-way ANOVA *P* value		
	Diet 1	Diet 2	Diet 6	Diet 1H	Diet 1	Diet 2	Diet 6	Diet 1H	Diet	Challenge	Diet* Challenge
ΣSFA	24.5 ± 0.9	25.2 ± 0.5	24.8 ± 0.4	25.4 ± 0.9	24.5 ± 0.8	23.8 ± 1.0	23.6 ± 0.8	25.5 ± 1.2	ns	ns	ns
ΣMUFA	34.3 ± 3.0^a^	30.6 ± 1.5^ac^	20.2 ± 1.2^b^	26.9 ± 2.1^c^	33.4 ± 2.8^a^	32.4 ± 2.8^a^	21.8 ± 0.3^b^	25.6 ± 1.4^bc^	<0.0001	ns	ns
18:2n-6	6.1 ± 0.5^a^	8.7 ± 1.5^b^	17.9 ± 0.8^c^	8.4 ± 1.1^d^	5.9 ± 0.6^a^	9.7 ± 0.9^b^	21.0 ± 0.7^e^	7.6 ± 0.9^d^	<0.0001	0.0103	0.0005
20:2n-6	0.7 ± 0.1^a^	0.9 ± 0.1^b^	1.9 ± 0.2^c^	0.9 ± 0.1^b^	0.7 ± 0.1^a^	1.0 ± 0.0^b^	1.8 ± 0.1^c^	0.8 ± 0.2^ab^	<0.0001	ns	ns
20:3n-6	1.4 ± 0.2^a^	1.9 ± 0.2^b^	2.2 ± 0.1^c^	0.7 ± 0.1^d^	1.4 ± 0.2^a^	1.6 ± 0.1^ab^	2.0 ± 0.2^c^	0.8 ± 0.1^d^	<0.0001	ns	ns
20:4n-6 (ARA)	3.4 ± 0.5^a^	4.3 ± 0.4^ab^	5.4 ± 0.6^c^	3.7 ± 0.5^ab^	3.8 ± 0.6^ab^	3.7 ± 0.5^ab^	4.7 ± 0.1^b^	4.1 ± 0.3^ab^	0.0002	ns	0.0137
Σn-6	11.9 ± 0.4^a^	16.3 ± 0.4^b^	27.2 ± 0.7^c^	14.2 ± 0.5^d^	12.2 ± 0.2^a^	16.4 ± 0.3^b^	30.6 ± 0.7^e^	13.7 ± 0.7^d^	<0.0001	0.0946	0.0006
18:3n-3	1.0 ± 0.2^a^	0.8 ± 0.4^a^	0.8 ± 0.1^a^	1.9 ± 0.4^b^	0.90 ± 0.2^a^	1.0 ± 0.2^a^	1.1 ± 0.1^a^	1.6 ± 0.4^b^	<0.0001	ns	ns
20:4n-3	0.4 ± 0.1^a^	0.3 ± 0.1^a^	0.3 ± 0.0^a^	0.5 ± 0.1^b^	0.4 ± 0.1^a^	0.4 ± 0.1^a^	0.2 ± 0.1^a^	0.5 ± 0.1^b^	<0.0001	ns	ns
20:5n-3 (EPA)	4.4 ± 0.5^a^	3.9 ± 0.3^a^	3.0 ± 0.3^b^	5.6 ± 0.6^c^	4.8 ± 0.4^a^	4.2 ± 0.2^a^	3.0 ± 0.2^b^	6.0 ± 0.5^c^	<0.0001	ns	ns
22:5n-3	1.1 ± 0.2^a^	1.1 ± 0.1^a^	1.0 ± 0.1^a^	1.5 ± 0.2^b^	1.2 ± 0.1^a^	1.2 ± 0.0^a^	0.9 ± 0.1^a^	1.5 ± 0.1^b^	0.0006	ns	ns
22:6n-3 (DHA)	16.9 ± 2.7^ab^	17.0 ± 1.2^ab^	16.5 ± 1.0^ab^	18.7 ± 1.7^b^	17.8 ± 1.7^a^	16.0 ± 2.0^a^	16.6 ± 0.3^a^	20.9 ± 1.7^b^	0.0005	ns	ns
Σn-3	24.4 ± 2.6^a^	23.7 ± 0.9^a^	21.9 ± 1.1^a^	29.0 ± 1.7^b^	25.9 ± 1.8^a^	23.3 ± 1.9^a^	22.2 ± 0.3^a^	30.9 ± 0.8^b^	<0.0001	ns	ns
n-6/n-3	0.50 ± 0.1^a^	0.7 ± 0.0^b^	1.3 ± 0.1^c^	0.5 ± 0.1^a^	0.5 ± 0.0^a^	0.7 ± 0.1b	1.4 ± 0.0^c^	0.4 ± 0.1^a^	<0.0001	ns	0.0116

**Notes:**

PGE_2_-EP4, prostaglandin E2-EP4 receptor; COX2, cyclooxygenase2; TNF-α, tumour necrosis factor- α; IL-1β, induction of interleukin-1β; iNOS, inducible nitric oxide synthase; IL413-b, interleukin 4/13b; GATA3, transcription factor GATA binding protein3; p53, tumor suppressor protein p53; Hsp70, Heat shock protein 70; Hsp90, Heat shock protein 90 ; β-actin, Beta actin; EF1 αb, Elongation factor 1 alpha B.

Results are means ± SD (for challenged group *n* = 3, for non-challenged group. three fish per diet). *p*-values of two-way ANOVA are presented for factors ‘diet’, ‘AGD challenge’ and interaction between diet and AGD challenge. ns, not statistically significant (*p* > 0.05). Different superscript letters within an individual row denote statistically significant differences in fatty acid content according to Tukey’s multiple comparison test.

FA, fatty acid; ΣSFA, sum of saturated fatty acids; ΣMUFA, sum of monounsaturated fatty acids; EPA, eicosapentaenoic acid; DHA, docosahexaenoic acid; PUFA, polyunsaturated fatty acids. Diet 1/Diet 2/Diet 6/Diet 1H, diet codes are set according to dietary n-6/n-3 ratio. The final diet is labelled 1H due to its higher absolute contents of n-3 and n-6 compared to the first diet.

### mRNA expression in gills

**Figure 4:**
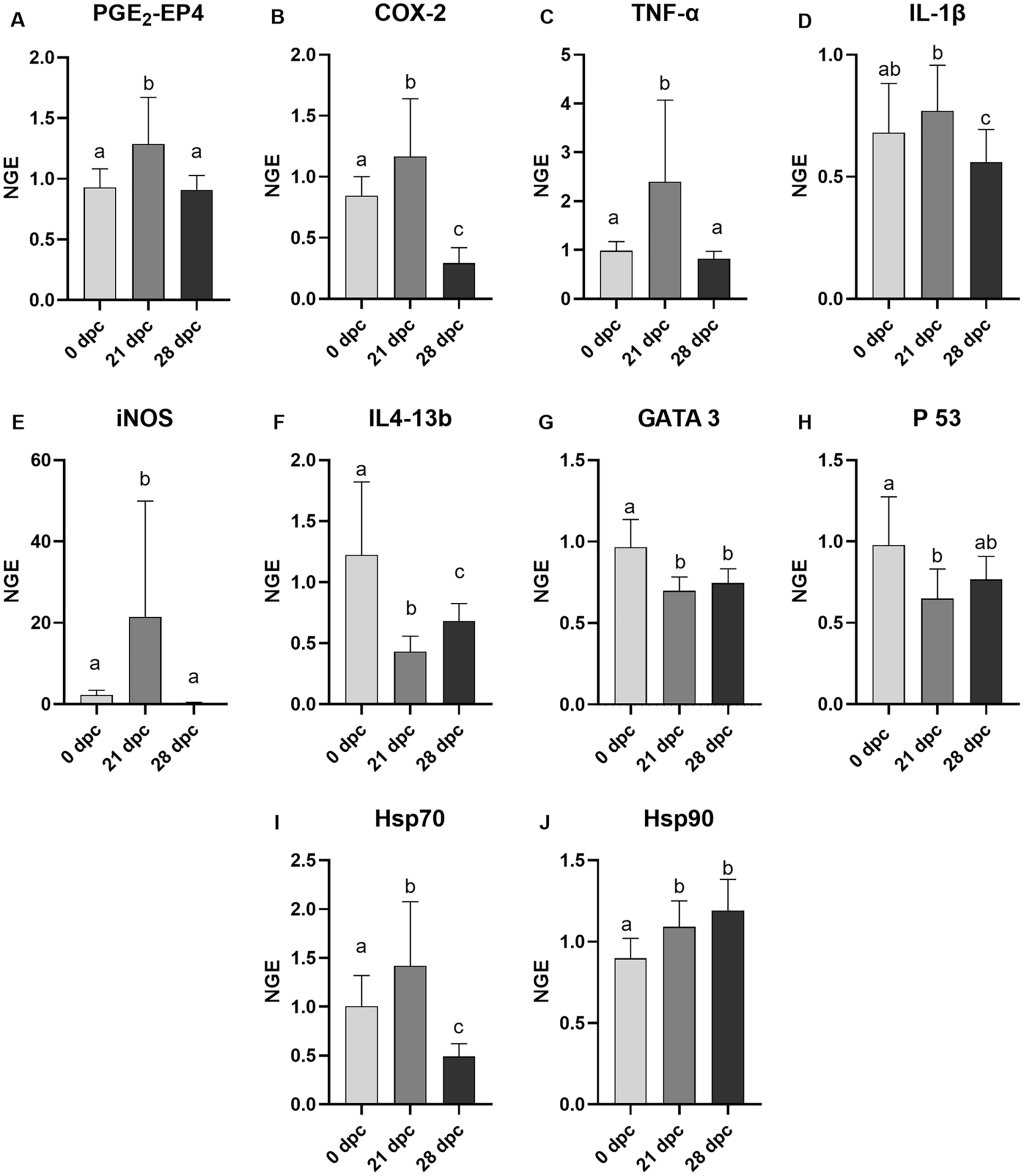
Mean normalized expression of gill mRNA from Atlantic salmon fed different level and ratio of n-6 and n-3 FA and challenged with *P. perurans*. Due to the lack of significant differences between dietary groups at any time point (Fig. S1), these were pooled in the figure to show the effects of the challenge. (A) PGE2-EP4, prostaglandin E2-EP4 receptor; (B) COX-2, cyclooxygenase2; (C) TNF-α, tumour necrosis factor- α; (D) IL-1β, induction of interleukin-1β; (E) iNOS, inducible nitric oxide synthase; (F) IL413-b, interleukin 4/13b; (G) GATA 3, transcription factor GATA binding protein; 3 (H) p53, tumor suppressor protein p53; (I) Hsp70, Heat shock protein 70; (J) Hsp90, Heat shock protein 90. Challenge effects were considered significant when *p* < 0.05 upon nested ANOVA followed by Tukey’s multiple comparison analysis between time-points. Different letters (a,b,c) represent significant difference between time-points. Data are presented as mean with standard deviation (*n* = 6/tank). Days of post-challenge, dpc.

The mRNA expression of the gills of the control and challenged fish were analyzed, and samples from 0 dpc (pre-challenge), 21 dpc (maximum gill score) and 28 dpc (recovery phase) samplings were used (Fig. 4). In general, AGD influenced the mRNA expression of examined genes involved in immune and inflammatory response (TNF-α, iNOS, IL4-13b, GATA-3, IL-1β, p53, COX2 and PGE_2_-EP4), and most of these genes were either upregulated or downregulated at 21 dpc compared to 0 dpc and 28 dpc. The mRNA expression of all these genes remained equivocal at the pre-challenge and recovery phase than compared to the maximum gill score phase. However, diets did not affect all the examined gene expression at 21 dpc (Fig. S1) so the dietary treatments are presented grouped in the Fig. 4. At 21 dpc (maximum gill score), the mRNA expression of TNF-α and iNOS were significantly upregulated (TNF-α, *p* = 0.0002; iNOS, *p* < 0.0001) when compared to both 0 dpc (pre-challenge) and 28 dpc (recovery phase). Similarly, expression of stress proteins Hsp70 and Hsp90 mRNA expression were also significantly upregulated at 21 dpc (Hsp70, *p* = 0.0005; Hsp90, *p* < 0.0001) when compared to 0 dpc (pre-challenge). However, at 28 dpc, Hsp90 mRNA expression remained elevated, whereas Hsp70 mRNA expression was significantly downregulated when compared to 0 dpc and 21 dpc. Expression of IL4-13b, GATA-3 (the transcriptional factors for IL4-13 cytokines), and p53 mRNA were significantly downregulated at 21 dpc when compared to 0 dpc and 28 dpc (IL4-13b, *p* < 0.0001; GATA-3, *p* = 0.0011; p53, *p* = 0.0088; Fig. 4). The IL-1β mRNA expression was significantly high at 21 dpc compared to 28 dpc; however, no difference was found between 0 dpc and 21 dpc. The mRNA expression of COX2 and EP4 (Prostaglandin E2 receptor 4) genes were also significantly upregulated (*p* < 0.0001) at 21 dpc compared to 0 dpc and 28 dpc.

## Discussion

A series of investigations have reported that substitution of fish oil with different vegetable oils or different dietary ratios n-6/n-3 FA does not affect the growth performance or robustness of Atlantic salmon (Bransden, Carter & Nichols, 2003; Gjøen et al., 2005; Glencross et al., 2014; Katan et al., 2019; Sissener et al., 2016b; Torstensen et al., 2005). Accordingly, two trials using the same diets as in the current work, reported no dietary effects on final weight of Atlantic salmon after a three-month growth trial in seawater (Hundal et al., 2020) or after a 4 weeks stress trial following the growth trial, involving both repeated and acute stress (Hundal et al., 2021). However, in the present study, the diet with highest n-6/n-3 ratio (diet 6, 6:1) negatively affected the growth of the fish, but only in the fish challenged with *P. perurans*. This reduction of weight gain in fish fed high n-6/n-3 FA ratio could be due to combined effects of diet, AGD and the added handling stress introduced by weekly sampling. Further, the hypothesis of increased stress in all *P. perurans* challenged fish is supported by increased mRNA expression of stress markers Hsp70 and Hsp90 genes in gills at 21 dpc (Fig. 4), concurrent with the peak of gross pathology scores in gills (Fig. 2). Thereby, the added factor of a sub-optimal dietary n-6/n-3 ratio could have added additional stress to the fish and thus causing the observed changes in growth. Although the present study was of relatively short duration and more focused to test the effects of FA on AGD development, the results could suggest that the dietary n-6/n-3 FA ratio may affect growth during an AGD challenge and is therefore an important factor to be considered. As Amoebic gill disease (AGD) is currently one of the major challenges for the Atlantic salmon farming industry (English et al., 2019; Oldham, Rodger & Nowak, 2016; Shinn et al., 2015), thus the use of diets with a high level of n-6 FA relative to n-3 FA should not be recommended, based on our results.

In general, dietary FA can either be incorporated into cell membranes (polar lipids, PLs) as phospholipids or be stored as reserve lipids (neutral lipids, NLs), mainly as triglycerides. In fish, as in mammals, the dietary FA composition greatly influences the membrane FA composition (Tocher, 2003). The three FAs ARA, EPA and DHA are considered crucial for cell membrane functions which includes membrane permeability, fluidity, membrane fusion and ion transportation. In addition, these FA also serve as precursors for production of immunologically active eicosanoids and resolvins, hence being important for proper health status (Arts & Kohler, 2009; Hagve, 1988; Valentine & Valentine, 2004). Any diet-induced changes in membrane FA compositions could directly influence the fish’s immune system and health (Bell et al., 1996b; Bell, Sargent & Raynard, 1992; Tocher et al., 2003). Interestingly, we found that fish fed a high dietary n-6/n-3 ratio (diet 6) had significantly reduced 18:2n-6 content in gills when challenged compared to the fish in non-challenged conditions. As there was also a significant interaction between diet and challenge for ARA, where ARA seemed to be affected in the opposite direction to 18:2n-6 in diet group 6 when the fish were challenged, this could suggest an increased conversion from 18:2n-6 to the eicosanoid precursor ARA in challenged fish. Eicosanoids levels were not analyzed in the current study, but a feeding trial in Atlantic salmon using the same diets, reported that the fish fed the high n-6/n-3 ratio diet (diet 6, 6:1) had significantly elevated liver PGE_2_ levels both before and after stressing conditions (Hundal et al., 2021). Changes in n-6 FA have been seen in earlier studies with a typical inflammatory response, such as in Sanden et al. (2018) where they reported that dietary pesticide chlorpyrifos-methyl affected the arachidonic acid metabolism in Atlantic salmon. They found a significantly reduced ARA content in response to increasing dietary pesticide exposure, indicating a possible change in eicosanoid synthesis from n-6 precursors.

Despite low gill scores and slow disease progression, the mRNA expression of selected immune and inflammatory genes were significantly influenced by the *P. perurans* infection. There was a demonstrable difference in gills mRNA expression of TNF-α, iNOS, PGE_2_-EP4 receptor, COX-2, IL4-13b, p53, GATA3, Hsp70 and Hsp90 during the challenge period. Notably, most of these genes were either up-regulated or down-regulated at 21dpc compared to the two other time points pre-infection and at a resolution stage. The expression of COX2 (enzyme that catalyzes the conversion of ARA to prostaglandins), inducible nitric oxide synthase (iNOS, key enzyme in NO synthesis) and PGE_2_-EP4 receptor were all significantly increased at 21 dpc compared to the two other time points sampled. Earlier studies with Atlantic salmon fed soybean oil diets, rich in n-6 FA, have shown induction of COX-2 expression in gill and intestinal tissue (Olsen et al., 2012; Oxley et al., 2010), as well as in head kidney leucocytes (Holen et al., 2018). There were, however, no significant dietary effects on the expression of these genes in the current trial. The EP4 receptor, one of four subtypes of the EP prostanoid receptor, is preferentially activated by prostaglandin E_2_ (PGE_2_) (Jones, 2007; Konya et al., 2013), thus indicating an increased presence of the ARA-derived PGE_2_ in the gills at this time point. In fish, the importance of induced iNOS expression and nitric oxide production as a host protective immune response against pathogen infection has been demonstrated (Acosta et al., 2004; Bridle, Morrison & Nowak, 2006). Further, the Co-expression of COX-2 and iNOS is also well documented (Chiarugi, Magnelli & Gallo, 1998; Rahman et al., 2001). Additionally, Timoshenko, Lala & Chakraborty (2004) demonstrated a PGE_2_-mediated upregulation of iNOS in murine breast cancer cells through the activation of EP4 receptor. Therefore, the upregulation of the mRNA expression of COX2, iNOS, and PGE_2_-EP4 at the same time point (21 dpc) where fish had the highest gross pathology score supports the hypothesis that there may have been an activation of the eicosanoid synthesis at this time point.

In the current study, an increased dietary n-6/n-3 ratio significantly reduced EPA levels in the gills but did not affect DHA levels. Neither FA were affected in fish exposed to *P. perurans*. The reduction of the EPA with increasing n-6/n-3 ratio occurred even when dietary EPA and DHA were unchanged. This is in line with previous results using the same diets (Hundal et al., 2020), where they reported reductions of EPA in liver polar lipids with increased dietary n-6/n-3 ratio. In general, the FA composition of fish gills is mostly composed of polar lipids (fat content typically ~2.2% wet weight; Fountoulaki et al., 2003) and the fat is therefore assumed to be mostly polar and reductions in EPA according to what Hundal et al. (2020) explained. In brief, it is generally accepted that DHA is preferentially incorporated over EPA into membrane phospholipids as a structural component (Brodtkorb, Rosenlund & Lie, 1997; Stillwell & Wassall, 2003). A decrease in EPA as reported might also be utilized to produce anti-inflammatory and inflammation resolving resolvins (Calder, 2010; Wall et al., 2010). A decreased EPA with increasing n-6/n-3 ratios in the diets may also be due to increased competition for enzymes shared by n-6 and n-3 FA. (Buzzi, Henderson & Sargent, 1996; Henderson & Tocher, 1987; Tocher, 2003). The result of this decreased membrane EPA in fish fed high n-6/n-3 ratio, could be a change in the overall inflammation status of the fish.

A clinical sign of AGD is the colonization of the gills by *P. perurans* and, consequently, the formation of white mucoid spots and plaques on the gill surface. These white spots are grossly assessed and scored on a scale of clear to heavy (Adams, Ellard & Nowak, 2004; Taylor et al., 2009b). In the present study, although no AGD lesions were observed histologically, gross gill scores were increased over time in the challenged fish and reached a maximum at 21 dpc, declining thereafter, and leading to the fish spontaneously recovering. Fish at 21 dpc showed a mild hyperplastic lesion (ht) with fused adjacent lamellae and a stratified layer of epithelial tissue at the lesions surface. Diets did not appear to have any effect on gill score or disease progression. This is in agreement with previous studies where increased dietary n-6/n-3 FA had no influence one disease resistance of Atlantic salmon against bacterial pathogens (Bransden, Carter & Nichols, 2003; Gjøen et al., 2005). However, this result also contradicts other studies, where it has been reported that high dietary n-6 FA negatively affects disease resistance against bacterial infection in Atlantic salmon (Thompson, Tatner & Henderson, 1996). In commercial aquaculture practice, a gill score of 2 and above is the point where intervention treatment would be started (Taylor et al., 2009b). However, in the present study, although there was a significant difference in gill scores over the challenge period, the maximum gill score was relatively low (only minor gill pathology was observed; Fig. 2) compared to many other studies (Marcos-Lopez et al., 2018; Marcos-Lopez et al., 2017; Rosenlund, 2017; Taylor et al., 2009b; Wiik-Nielsen et al., 2016). Similar reduced gill scores have been reported previously by Bridle et al. (2005); however, unlike the present study, amoebae were found on the gills of the challenged fish. Moreover, they observed an enhanced survival of a sub-population of Atlantic salmon exposed to *P. perurans* with relatively minor gill pathology. The reason for this may be complex and a combination of acquired resistance, be it immunological or genetic in origin (Robledo et al., 2020; Robledo et al., 2018; Taylor et al., 2009a; Taylor et al., 2007; Findlay & Munday, 1998; Findlay et al., 1995; Vincent, Morrison & Nowak, 2006).

Macrophages are essential for host defense (Mosser & Edwards, 2008; Rolot & Dewals, 2018; Van Dyken & Locksley, 2013). In general, macrophages are activated in number of ways, including interaction with immune cells such as CD8+, NK cells, and other monocytes (eosinophils, and basophils), macrophages and pre-exposed to a diversity of PAMPs (pathogen associated molecular patterns) (Jang & Nair, 2013; Martinez & Gordon, 2014; Mills et al., 2000). However, recent evidence from AGD experiments (Benedicenti et al., 2015; Marcos-Lopez et al., 2018) suggests that the classical Th1-type pathway or the alternate Th2-type pathway are most likely involved during the development of *P. perurans* infection. In any case, pro-inflammatory cytokines, including those from Th1-type cells such as IFN-γ and TNF-α induce iNOs that stimulate the NO production by granulocytes are released. Alternatively, cytokines such as IL4/13, IL-1β are released and needed the Th2-type pathway. Previous studies in salmon have shown that there is a switching from Th1 to Th2-type pathway in gills as AGD progresses (Benedicenti et al., 2015; Marcos-Lopez et al., 2018). A similar a switching from Th1 to Th2 pathway in response to sea lice infection has also been reported in Coho salmon (*Oncorhynchus kisutch*) (Braden, Koop & Jones, 2015) and rainbow trout (*O. mykiss*) (Chettri et al., 2014). They found that an early pro-inflammatory Th1-type pathway as an initial host response during infection with Pacific Sea lice and demonstrates subsequent regulatory Th2-type processes as candidate defense mechanisms as disease progresses. In the present study, the mRNA expression of Th1 pro-inflammatory cytokines such as TNF-α and the enzymes iNOS were markedly increased at the time point of maximum gill scores (21 dpc), while the Th2 pro-inflammatory cytokine IL4-13b was downregulated at the same time. GATA-3 (the transcriptional factor for IL4-13 cytokines) was also significantly downregulated at 21 dpc compared to the pre-challenge and recovery phase. These results suggest that an activation of the Th1 pathway is an initial host response and also more prominent during a low-grade/early stage AGD. This contradicts the work of Rosenlund (2017), where the opposite was found where AGD was more severe. IL-1β has been identified as one of the hallmarks of Atlantic salmon response to AGD (Nowak et al., 2014). In the present study, IL-1β expression was significantly upregulated at the maximum gill score compared to in the recovery phase. Further, there was a clear downregulation of tumor suppressor protein (p53), a well-studied protein involved in the cellular stress response pathway (Guo et al., 2016) at the maximum gill score phase (21 dpc) compared to pre-challenge (0 dpc) and recovery phase (28 dpc). This is in agreement with previous reports, where a significant downregulation of p53 expression is described as one of the underlying mechanisms for cell proliferation in AGD (Morrison et al., 2006). The elevated mRNA expression of stress-related genes Hsp90 and Hsp70 in the present study is also in agreement with previous results (Marcos-López et al., 2017), where they are suggested to be involved indirectly in cell proliferation. Taken together, this demonstrated gene expression in gills has provided novel evidence of a Th1 type immune response to a low-grade/early stage AGD compared to other studies where AGD is more severe.

The reports on the effect of dietary n-6 FA levels on the fish immune system are contradictory, ranging from no apparent dietary effects (Andresen et al., 2019; Bransden, Carter & Nichols, 2003; Gjøen et al., 2005), as seen in the present study, to reduced resistance against or increased mortality from pathogens (Thompson, Tatner & Henderson, 1996; Martinez-Rubio et al., 2012; Estensoro et al., 2012; Montero et al., 2008; Montero et al., 2010; Montero et al., 2019). Furthermore, it has been suggested that other dietary nutrients such as the sources of protein, availability of micronutrients, species differences, and experimental conditions may all impact the immune response.

## Conclusion

The result of the present study showed that dietary n-6 and n-3 FA or their ratios were ineffective in altering the mRNA expression of immune genes or the progression of the disease in Atlantic salmon challenged with *P. perurans*. However, a high n-6/n-3 ratio of 6 caused a significant reduction in growth during a *P. perurans* challenge and subsequent development of low-grade hyperplastic lesions, compared to fish fed diets with low n-6/n-3 ratios at 1 and 2. Therefore, this is an important factor to be considered when formulating Atlantic salmon diets. Additionally, our study indicates the activation of Th1 type immune response to low-grade hyperplastic lesions and reports the spontaneous recovery of fish.

## Supplemental Information

10.7717/peerj.12028/supp-1 Supplemental Information 1 Raw data: Mean normalized expression of gill mRNA from Atlantic salmon challenged with *N. perurans*.

10.7717/peerj.12028/supp-2 Supplemental Information 2 Raw data: Fatty acid composition (percentage of total fatty acids) of gill tissue from Atlantic salmon fed the different diets at 21 days of post challenge with AGD.

10.7717/peerj.12028/supp-3 Supplemental Information 3 Raw data: Weight (A) and Length (B) and gill score of challenged fish during challenge period. Data presented as mean with standard deviation (*n* = 3).

10.7717/peerj.12028/supp-4 Supplemental Information 4 Mean normalized expression of gill mRNA from Atlantic salmon fed different level and ratio of n-6 and n-3 FA and challenged with challenged with *P. perurans*.. (A) PGE2-EP4, prostaglandin E2-EP4 receptor; (B) COX2, cyclooxygenase2; (C) TNF- α , tumour necrosis factor- α; (D) IL-1 β , induction of interleukin-1β; (E) iNOS, inducible nitric oxide synthase; (F) IL413-b, interleukin 4/13b; (G) GATA-3, transcription factor GATA binding protein; 3) (H) p53, tumor suppressor protein p53; (I) HSP 70, Heat shock protein 70; (J) HSP 90, Heat shock protein 90. Challenge effects were considered significant when *p* < 0.05 upon nested ANOVA followed by Tukey’s multiple comparison analysis between time-points. Different letters (a, b, c) represent significant difference between time-points. No significance difference between dietary groups were detected at any of the time points. Data presented as mean with standard deviation (*n* = 6/tank). Diet 1/Diet 2/Diet 6/Diet 1H, diet codes are set according to dietary n-6/n-3 ratio. The final diet is labelled 1H due to its higher absolute contents of n-3 and n-6 compared to the first diet.

10.7717/peerj.12028/supp-5 Supplemental Information 5 Final weight and length of Atlantic salmon fed different level and ratio of n-6 and n-3 FA and challenged with *P. perurans*. . Data presented as mean with standard deviation (*n* = 3). Two-way ANOVA followed by Tukey’s Multiple comparison was performed for factors diet and AGD challenge.

10.7717/peerj.12028/supp-6 Supplemental Information 6 Gill score of Atlantic salmon fed different level and ratio of n-6 and n-3 FA and challenged with *P. perurans*. Data presented as mean with standard deviation (*n* = 3). Two-way ANOVA followed by Tukey’s Multiple comparison was performed for factors diet and AGD challenge.

10.7717/peerj.12028/supp-7 Supplemental Information 7 ARRIVE 2.0 checklist.

## Additional Information and Declarations

### Data Availability

The following information was supplied regarding data availability: Mean normalized expression of gill mRNA from Atlantic salmon fed different diets challenged with N. perurans and raw data are available in the Supplemental Files.
