# Supplementary figures and images for: Impact of dietary level and ratio of n-6 and n-3 fatty acids on disease progression and mRNA expression of immune and inflammatory markers in Atlantic salmon (Salmo salar) challenged with Paramoeba perurans
Source: PeerJ. 2021 Aug 31;9:e12028. doi: 10.7717/peerj.12028 (PMC8415286; doi:10.7717/peerj.12028)

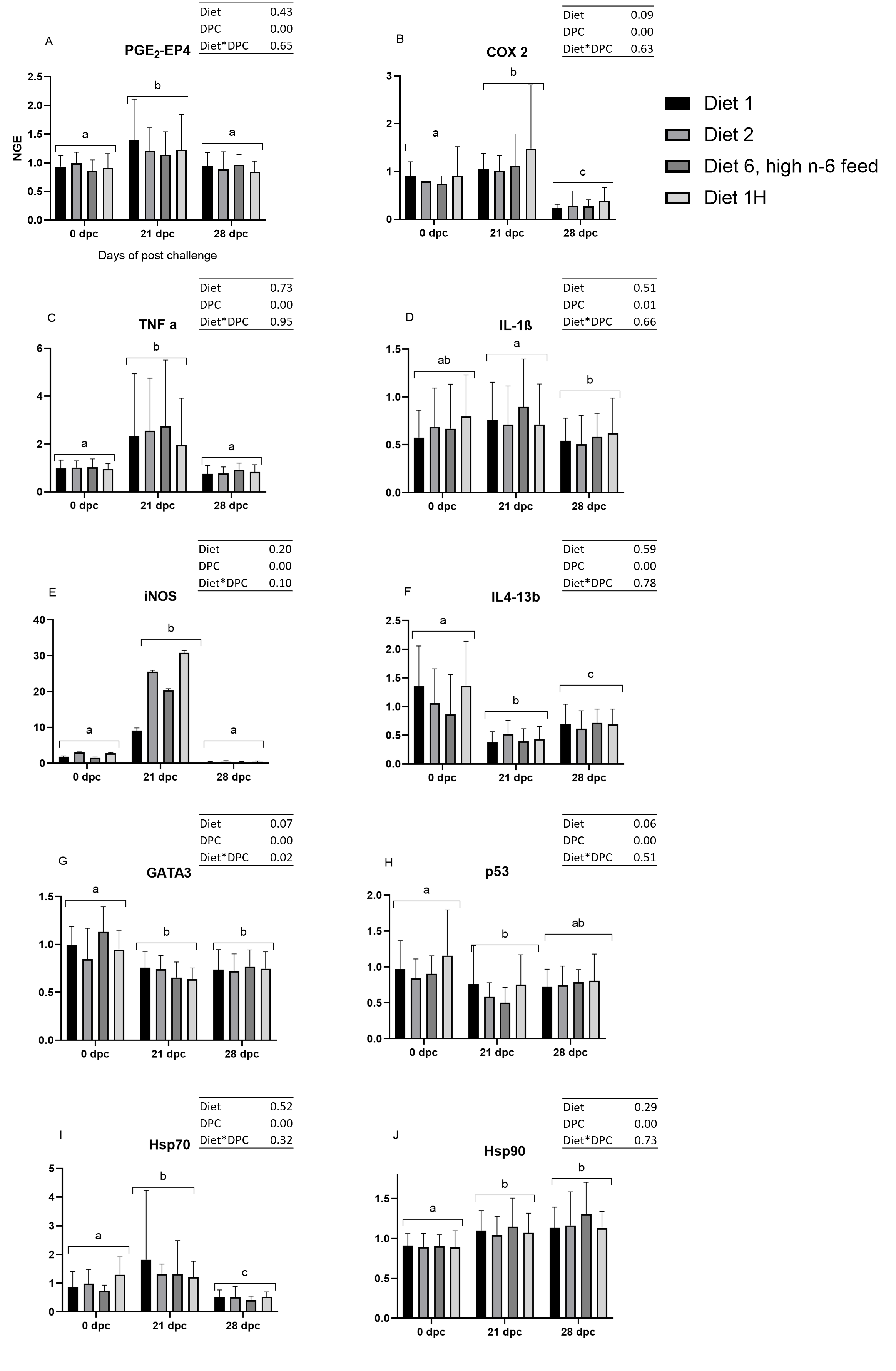

Supplement: Supplemental Information 4 — (A) PGE2-EP4, prostaglandin E2-EP4 receptor; (B) COX2, cyclooxygenase2; (C) TNF- α , tumour necrosis factor- α; (D) IL-1 β , induction of interleukin-1β; (E) iNOS, inducible nitric oxide synthase; (F) IL413-b, interleukin 4/13b; (G) GATA-3, transcription factor GATA binding protein; 3) (H) p53, tumor suppressor protein p53; (I) HSP 70, Heat shock protein 70; (J) HSP 90, Heat shock protein 90. Challenge effects were considered significant when p < 0.05 upon nested ANOVA followed by Tukey’s multiple comparison analysis between time-points. Different letters (a, b, c) represent significant difference between time-points. No significance difference between dietary groups were detected at any of the time points. Data presented as mean with standard deviation (n = 6/tank). Diet 1/Diet 2/Diet 6/Diet 1H, diet codes are set according to dietary n-6/n-3 ratio. The final diet is labelled 1H due to its higher absolute contents of n-3 and n-6 compared to the first diet. [file peerj-09-12028-s004.png]
