# Supplementary material for: Impact of dietary level and ratio of n-6 and n-3 fatty acids on disease progression and mRNA expression of immune and inflammatory markers in Atlantic salmon (Salmo salar) challenged with Paramoeba perurans
Source: PeerJ. 2021 Aug 31;9:e12028. doi: 10.7717/peerj.12028 (PMC8415286; doi:10.7717/peerj.12028)
Supplement: Supplemental Information 5 — Data presented as mean with standard deviation (n = 3). Two-way ANOVA followed by Tukey’s Multiple comparison was performed for factors diet and AGD challenge. [file peerj-09-12028-s005.docx]

**Supplementary table 1: Final weight and length of Atlantic salmon fed different level and ratio of n-6 and n-3 FA and challenged with *P. perurans***. Data presented as mean with standard deviation (n=3). Two-way ANOVA followed by Tukey’s Multiple comparison was performed for factors diet and AGD challenge.

|  | Diet 1 | | Diet 2 | | Diet 6 | | Diet 1H | | Two-way ANOVA P value | | |
| --- | --- | --- | --- | --- | --- | --- | --- | --- | --- | --- | --- |
|  | Mean | SD | Mean | SD | Mean | SD | Mean | SD | Diet | Challenge | Diet*challenge |
| Final weight | 467.62 | 75.43 | 483.40 | 67.32 | 405.89 | 86.23 | 454.80 | 76.34 | <0.0001 | <0.0001 | 0.0712 |
| Final Length | 31.56 | 1.00 | 32.11 | 0.55 | 30.24 | 0.80 | 31.85 | 0.57 | <0.0001 | <0.0001 | 0.4770 |
