# Supplementary material for: Impact of dietary level and ratio of n-6 and n-3 fatty acids on disease progression and mRNA expression of immune and inflammatory markers in Atlantic salmon (Salmo salar) challenged with Paramoeba perurans
Source: PeerJ. 2021 Aug 31;9:e12028. doi: 10.7717/peerj.12028 (PMC8415286; doi:10.7717/peerj.12028)
Supplement: Supplemental Information 6 — Data presented as mean with standard deviation (n = 3). Two-way ANOVA followed by Tukey’s Multiple comparison was performed for factors diet and AGD challenge. [file peerj-09-12028-s006.docx]

Supplementary table 2: **Gill score of Atlantic salmon fed different level and ratio of n-6 and n-3 FA and challenged with *P. perurans***. Data presented as mean with standard deviation (n=3). Two-way ANOVA followed by Tukey’s Multiple comparison was performed for factors diet and AGD challenge.

|  | Diet 1 | | Diet 2 | | Diet 6 | | Diet 1H | | AVG control | |
| --- | --- | --- | --- | --- | --- | --- | --- | --- | --- | --- |
|  | Mean | SD | Mean | SD | Mean | SD | Mean | SD | Mean | SD |
| 0 dpc | 0.06 | 0.25 | 0.16 | 0.37 | 0.09 | 0.39 | 0.28 | 0.63 | 0.16 | 0.45 |
| 7 dpc | 0.33 | 0.61 | 0.53 | 0.57 | 0.53 | 0.57 | 0.67 | 0.71 | 0.43 | 0.64 |
| 14 dpc | 0.93 | 0.78 | 0.80 | 0.61 | 0.80 | 0.61 | 0.90 | 0.61 | 0.38 | 0.70 |
| 21 dpc | 1.17 | 0.64 | 1.27 | 0.61 | 1.33 | 0.85 | 1.10 | 0.74 | 0.50 | 0.78 |
| 28 dpc | 0.67 | 0.80 | 0.48 | 0.60 | 0.50 | 0.61 | 0.48 | 0.68 | 0.09 | 0.29 |
| 35 dpc | 0.57 | 0.75 | 0.45 | 0.60 | 0.45 | 0.47 | 0.70 | 0.62 |  |  |

Two-way ANOVA P value for diet 0.7465; challenge p= <0.0001; Diet*challenge= 0.9981
